# Thermal reaction norms of key metabolic enzymes reflect divergent physiological and behavioral adaptations of closely related amphipod species

**DOI:** 10.1038/s41598-021-83748-2

**Published:** 2021-02-25

**Authors:** Lena Jakob, Kseniya P. Vereshchagina, Anette Tillmann, Lorena Rivarola-Duarte, Denis V. Axenov-Gribanov, Daria S. Bedulina, Anton N. Gurkov, Polina Drozdova, Maxim A. Timofeyev, Peter F. Stadler, Till Luckenbach, Hans-Otto Pörtner, Franz J. Sartoris, Magnus Lucassen

**Affiliations:** 1 Department of Integrative Ecophysiology, Alfred Wegener Institute Helmholtz Centre for Polar and Marine Research, Am Handelshafen 12, 27570 Bremerhaven, Germany; 2 Institute of Biology, Irkutsk State University, Karl Marx str.1, 664003 Irkutsk, Russia; 3 Bioinformatics Group, Department of Computer Science, University Leipzig, Leipzig, Germany; 4 Department of Bioanalytical Ecotoxicology, UFZ – Helmholtz Centre for Environmental Research, Permoserstr. 15, 04318 Leipzig, Germany; 5 LIFE, Leipzig Research Center for Civilization Diseases, University Leipzig, Leipzig, Germany; 6 Interdisciplinary Center for Bioinformatics, University Leipzig, Härtelstraße 16-18, 04107 Leipzig, Germany; 7 Max Planck Institute for Mathematics in the Sciences, Leipzig, Germany; 8 Department of Theoretical Chemistry, University of Vienna, Wien, Austria; 9 Facultad de Ciencias At, Universidad National de Colombia, Bogota, Colombia; 10 Santa Fe Institute, Santa Fe, NM USA

**Keywords:** Physiology, Metabolism, Energy metabolism

## Abstract

Lake Baikal is inhabited by more than 300 endemic amphipod species, which are narrowly adapted to certain thermal niches due to the high interspecific competition. In contrast, the surrounding freshwater fauna is commonly represented by species with large-scale distribution and high phenotypic thermal plasticity. Here, we investigated the thermal plasticity of the energy metabolism in two closely-related endemic amphipod species from Lake Baikal (*Eulimnogammarus verrucosus*; stenothermal and *Eulimnogammarus cyaneus*; eurythermal) and the ubiquitous Holarctic amphipod *Gammarus lacustris* (eurythermal) by exposure to a summer warming scenario (6–23.6 °C; 0.8 °C d^−1^). In concert with routine metabolic rates, activities of key metabolic enzymes increased strongly with temperature up to 15 °C in *E. verrucosus*, whereupon they leveled off (except for lactate dehydrogenase). In contrast, exponential increases were seen in *E. cyaneus* and *G. lacustris* throughout the thermal trial (Q_10_-values: 1.6–3.7). Cytochrome-c-oxidase, lactate dehydrogenase, and 3-hydroxyacyl-CoA dehydrogenase activities were found to be higher in *G. lacustris* than in *E. cyaneus*, especially at the highest experimental temperature (23.6 °C). Decreasing gene expression levels revealed some thermal compensation in *E. cyaneus* but not in *G. lacustris*. In all species, shifts in enzyme activities favored glycolytic energy generation in the warmth. The congruent temperature-dependencies of enzyme activities and routine metabolism in *E. verrucosus* indicate a strong feedback-regulation of enzymatic activities by whole organism responses. The species-specific thermal reaction norms reflect the different ecological niches, including the spatial distribution, distinct thermal behavior such as temperature-dependent migration, movement activity, and mating season.

## Introduction

Lake Baikal, an ancient freshwater ecosystem with long independent evolution (age: 25–30 million years), is inhabited by highly specialized and mostly endemic species colonizing all available ecological niches^1^. This high speciation suggests specific adaptations of the species to a narrow range of conditions in Lake Baikal and implies sensitivity to relatively slight deviations from the prevailing environmental conditions, such as a specific thermal regime. The peculiarities of Lake Baikal, the largest unfrozen freshwater body on earth, include stable high oxygen levels (12 mg L^−1^)^2^, low mineralization (~ 96 mg L^−1^)^3^, long seasonal ice-coverage (4–5 months), and intensive hydrodynamic mixing^4^. Therefore, average monthly temperatures in summer do usually not exceed 15 °C in the littoral zone at water depths of 0.5–1.5 m in Bolshie Koty, the sampling site of this study, although maximum temperatures of up to 20 °C may occur^5^. Lake Baikal’s particular abiotic and biotic conditions have remained stable for the last 2–4 million years^6^.

The taxonomic diversity of Lake Baikal’s fauna is exceptionally high; for example, Lake Baikal is inhabited by about 350 endemic amphipod species and subspecies that are dominant members of the littoral community in terms of species numbers and biomass^1,7^. In contrast, the surrounding freshwater fauna is mostly represented by amphipod species with broad ecological tolerance limits, high phenotypic plasticity, and large-scale distribution. A typical representative of this fauna is the freshwater amphipod *Gammarus lacustris* Sars, 1863, which has a nearly circum-boreal distribution that also extends into Southern Europe and Central Asia^8^.

Temperature is the most pervasive abiotic factor for animal life as it affects molecular dynamics and biochemical reaction rates^9^. The degree of phenotypic thermal plasticity classifies stenothermal and eurythermal organisms. Here, we aim at differentiating the thermal plasticity of two closely related amphipod species endemic to Lake Baikal, *Eulimnogammarus verrucosus* (Gerstfeldt, 1858), and *Eulimnogammarus cyaneus* (Dybowski, 1874), and *G. lacustris*, which also can be found in isolated bays of Lake Baikal. The examined endemic Baikal species inhabit overlapping spatial niches and are highly abundant in the upper littoral of Lake Baikal^10^. *Eulimnogammarus cyaneus* concentrates in the littoral zone at a depth of 0–1.5 m, where considerable temperature fluctuations and the lake’s highest maximum temperatures occur^5^. *Eulimnogammarus verrucosus* inhabits a water depth of 0–25 m^10^. In contrast to *E. cyaneus*, adult individuals of *E. verrucosus* retreat from the upper littoral to deeper and cooler waters when temperatures rise in summer in the littoral of Lake Baikal; this indicates a higher level of stenothermy for *E. verrucosus* compared to its relative *E. cyaneus*^11,12^. *Gammarus lacustris* is a very versatile species that can be found in deep and shallow lakes and slow-moving rivers in the Holarctic exhibiting large seasonal temperature fluctuations^13^.

Our previous studies revealed thermal limitations at the whole animal level by determining constraints in routine metabolism and ventilation activity of *E. verrucosus*, *E. cyaneus*, and *G. lacustris*^12,32^. Here, critical temperatures (Tc), determined by limitations in oxygen consumption^14^, were detected in the range of 14–15 °C for adult *E. verrucosus*, whereas the Tc for *E. cyaneus* and *G. lacustris* was at 23–25 °C^12,32^. Ventilation was limited at lower temperatures than routine metabolic rates (i.e., at 10–11 °C, 19–21 °C, and 21–22 °C in *E. verrucosus*, *E. cyaneus,* and *G. lacustris*, respectively) and exceeding these thermal thresholds likely goes along with reduced Darwinian fitness^12^. The amphipods were exposed to an experimental setup with gradually increasing temperature (0.8 °C per day), mimicking a summer warming scenario in Lake Baikal’s upper littoral. These maximum increases already observed in the field will likely occur more frequently in line with the prospected climate warming scenario, as the Baikal littoral is and will be particularly affected by climate change, causing the strongest effects in summer and autumn^15,16^. Here, we investigated the central metabolism of the three species underlying the oxygen consumption and ventilation rates determined in our previous study^12^. To get the best possible sketch of key metabolic processes, we quantified maximum enzyme activities of key metabolic enzymes, such as citrate synthase (CS), cytochrome-c-oxidase (COX), pyruvate kinase (PK), lactate dehydrogenase (LDH), hydroxy acyl CoA dehydrogenase (HADH) and glutamate dehydrogenase (GDH). We also quantified the respective gene expression levels and the transcription levels of about 300 ribosomal protein genes.

Against the background of climate change, this study aimed to assess (1) to what extent key metabolic processes of *E. verrucosus* (endemic to Lake Baikal; stenothermal) are adapted to a narrow thermal window, (2) whether *E. cyaneus* (endemic to Lake Baikal; eurythermal) and *G. lacustris* (ubiquitous in the Holarctic; eurythermal) show species-specific metabolic alterations despite their similar thermal tolerance and, (3) whether metabolic fuel use changes with rising temperatures in the three species.

## Material and methods

### Field sampling

**Figure 1:**
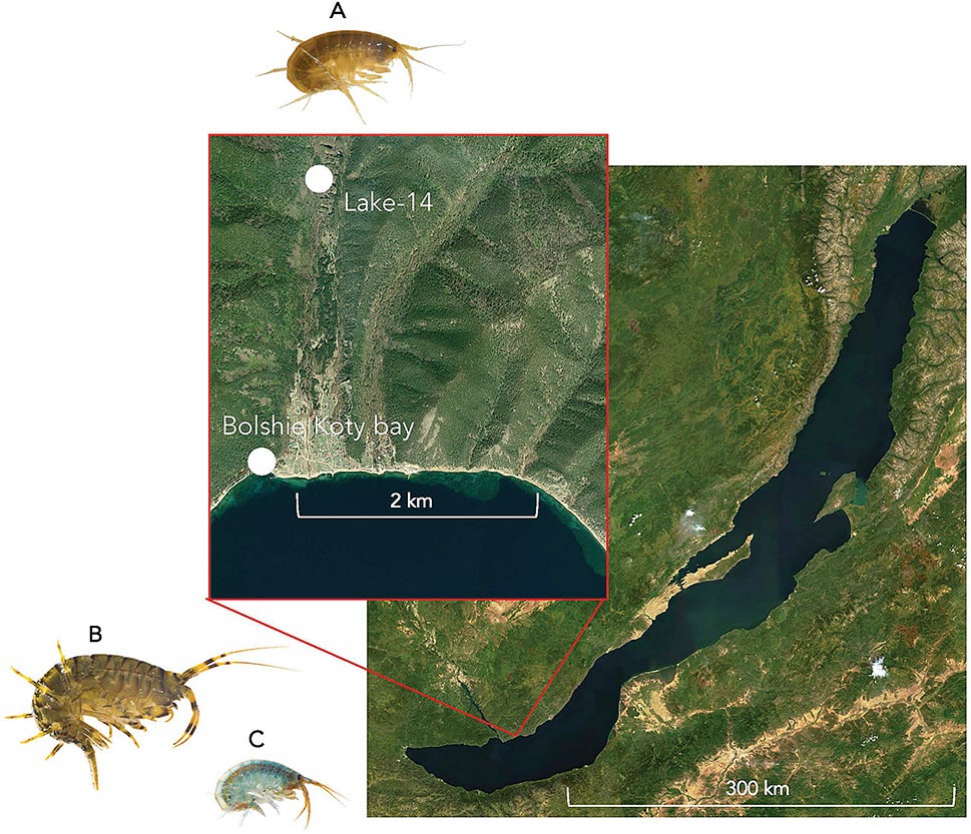
Sampling sites at Lake Baikal. *Gammarus lacustris* Sars,1863 (**A**) was sampled in a small shallow artificial water body named Lake-14 (51° 55′ 14.39′′ N, 105° 4′ 19.48′′ E). The Baikal species *Eulimnogammarus verrucosus* (Gerstfeldt, 1858) (**B**) and *Eulimnogammarus cyaneus* (Dybowski, 1874) (**C**) were sampled at the shoreline of Lake Baikal, close to Bolshie Koty (51° 54′ 11.67′′ N, 105° 4′ 7.61′′ E). The aerial maps were prepared using ArcGIS Online (www.arcgis.com) and are based on data from Esri (www.esri.com) and their data providers: USGS, NGA, NASA, CGIAR, GEBCO, N Robinson, NCEAS, NLS, OS, NMA, Geodatastyrelsen, the GIS User Community, Earthstar Geographics. The photographs of the amphipods were taken by Michael Ginzburg.

Adult animals (Fig. 1) were selected with a similar or larger size than those of previous studies, in which body length was used to classify adult animals^17^. In this study, *E. verrucosus*, *E. cyaneus*, and *G. lacustris* individuals with a fresh weight of about 550–850 mg (body length > 3 cm), 20–45 (body length ≅ 1 cm), and 30–150 mg (body length ≥ 1 cm) were used in the experiments. As *G. lacustris* shows a strong sexual dimorphism, with males being much larger than females^13^, the size range was relatively large for this species.

The sampling of the endemic amphipod species *E. verrucosus* and *E. cyaneus* was conducted with a hand net in the Baikal littoral (0–1.2 m depth) in the vicinity of Bolshie Koty (51° 9137″ N, 105° 0691″ E) by kick-sampling. The sampling site is part of the beach zone according to underwater landscape zoning ^10^, which is characterized by algae-covered boulders and intensive hydrodynamics. *Gammarus lacustris* was sampled in “Lake-14″ (51° 55′ 14.39″ N, 105° 4′ 19.48″ E), a eutrophic pond in about 2 km distance to Lake Baikal. This artificial water body is inhabited by species widespread across the Palearctic and Holarctic. It is connected to a backwater of Bolshie Koty river and supplied by groundwater. A map with the sampling locations is shown in Fig. 1.

Animals were sampled in late August, and early September, 2013, and sampling temperatures ranged from about 9–11.5 °C in Lake Baikal and from about 11–13 °C in Lake-14. Individuals of all three species were sampled in parallel.

## Experimental setup and animal maintenance

Animal incubations were performed in the Institute of Biology at Irkutsk State University (Irkutsk, Russia) in September 2013. The amphipods were transported to the laboratory in insulated boxes, sorted under temperature-controlled conditions, and kept in 2-L tanks (high-density polypropylene of food-grade quality or glass) filled with continuously aerated water from Lake Baikal with the temperature set to 6 °C, which is the average temperature of the littoral of Lake Baikal^18–20^. Maximum amounts of individuals per container were 200, 75 and 25 for *E. cyaneus*, *G. lacustris*, and *E. verrucosus*, respectively. Per species, six to ten tanks were set up. The animals were kept for at least three days in the tanks before starting the experiment to check whether all animals were intact. Due to coastal upwelling and weather changes, amphipods in the field are exposed to large and rapid temperature fluctuations (up to 13 °C within 24 h; personal observation). Therefore, we decided not to pre-acclimate the experimental animals at a constant temperature over long periods of time. Instead, we kept animals at a constant temperature of 6 ± 0.8 °C in a laboratory refrigerator during the entire experimental period to serve as parallel time control. Small clean pebbles on the tank bottoms served to provide shelter to the animals. Direct light was prevented in the tanks by opening the incubators only at dimmed light in the lab. Water was exchanged every one to three days, with increasing rates of water change at higher temperatures. A mix of amphipods, algae, water plants, and detritus collected in the Baikal littoral (frozen, air-dried at ≈ 30 °C and roughly mortared) was provided as food (*ad libitum*). The water temperature was controlled by keeping the tanks in an incubator (Sanyo MIR-254 (238 L), Osaka, Japan). The water temperature increase was set to a rate of 0.8 °C d^−1^. At each sampling time point, individuals of each species were randomly collected from the tanks, flash-frozen in liquid nitrogen, and stored at − 80 °C until analysis. Control animals were sampled at four different time points, at the start of the experiment (6 °C) and in parallel to samplings at 12.4 °C, 18.8 °C, and 23.6 °C of the temperature increase treatment. The sampling temperatures were chosen to cover the whole summer season. Temperatures of 5–6 °C usually prevail for several days in a row in early summer (June). In July, temperature rises at an unsteady rate (up to 0.8 °C h^−1^). The intermediate sampling temperature of 12.4 °C is in the optimum range for the two more eurythermal species *E. cyaneus* and *G. lacustris*. However, as our previous studies showed that temperatures ≥ 12 °C are already slightly stressful for *E. verrucosus* and that its routine metabolic rates leveled off at 15 °C^12,21^, we conducted additional samplings of the cold-stenothermal species *E. verrucosus* at 9.2 °C and 15.6 °C. A temperature of 18.8 °C can be considered a typical recent maximum temperature as up to 20 °C can be reached in July or August in the littoral at the study site. To take future warming into account, we increased the temperature up to 23.6 °C to slightly exceed current maximum summer temperatures.

### Enzyme assays

Measurements of enzyme activities were studied in homogenized tissue samples. For *E. cyaneus* and *G. lacustris* samples, seven and two to three individuals, respectively, were pooled due to the low weight of individual animals. Flash-frozen individuals were crushed with a mortar under continuous cooling with liquid nitrogen to prepare aliquots (60–100 mg). Subsequently, the tissue aliquots were homogenized with a Heidolph Brinkmann Tool 18F for Silent Crusher M (Fisher Scientific, Germany) at 16,500 rpm in ice-cold 20 mM Tris HCl buffer (pH 8.0), containing 1 mM EDTA, 0.1% TritonX100, and 100 mM NaCl. To prevent protein degradation, the Protease Inhibitor Cocktail powder P2714 (Sigma-Aldrich, Steinheim, Germany) was added as described in the manufacturer’s manual. The ratio of extraction buffer to tissue was 10 μL to 1 mg of tissue. Homogenization was performed on ice by using three 10-s homogenization intervals interrupted by 10-s breaks. Cell debris was removed by centrifugation (10 min; 4 °C; 1,000 × g) and the supernatant was used as a crude extract. Under optimized assay conditions, COX, CS, LDH, PK, HADH and GDH activities were measured in a temperature-controlled spectrophotometer system (SPECORD S600, Analytic Jena, Germany).

To study the thermal effect of incubation (sampling) temperature and assay (measuring) temperature on enzyme activities, maximum enzyme activities were determined at the respective sampling temperatures and additionally at 6 °C, 18.8 °C, and 23.6 °C. Samples taken from the 6 °C time control were measured at 6 °C and 18.8 °C. As buffer pH values may vary at different temperatures, they were previously adjusted to the respective measuring temperatures. UV-micro cuvettes (70–550 µL; Brand, Wertheim, Germany) were used as reaction vessels; the final volume was 200 µL. All parameters and components of the enzyme assays are summarized in Table S1 (Supplement). The assays were modified according to Sidell et al*.*^22^ (CS), Moyes et al.^23^ (COX), McClelland et al*.*^24^ (HADH), Sanchez-Muros et al.^25^ (GDH), Driedzic and De Almeida-Val^26^ (PK), and Kornberg^27^ (LDH), respectively. After ensuring thermal equilibration of all components resulting in a stable absorbance signal, the reactions were induced by adding the start reagents. Maximum enzyme activities were based on tissue wet weight. Protein concentrations of the tissue samples were determined by standard methods^28^ with bovine serum albumin as protein standard (Sigma-Aldrich, Steinheim, Germany). As the protein contents in the tissue extracts of the animals sampled at different temperatures did not vary significantly (ANOVA; p > 0.05, data not displayed) and whole animal parameters are also based on fresh weight, enzyme capacities presented in this study are based on tissue fresh weight.

**Figure 2:**
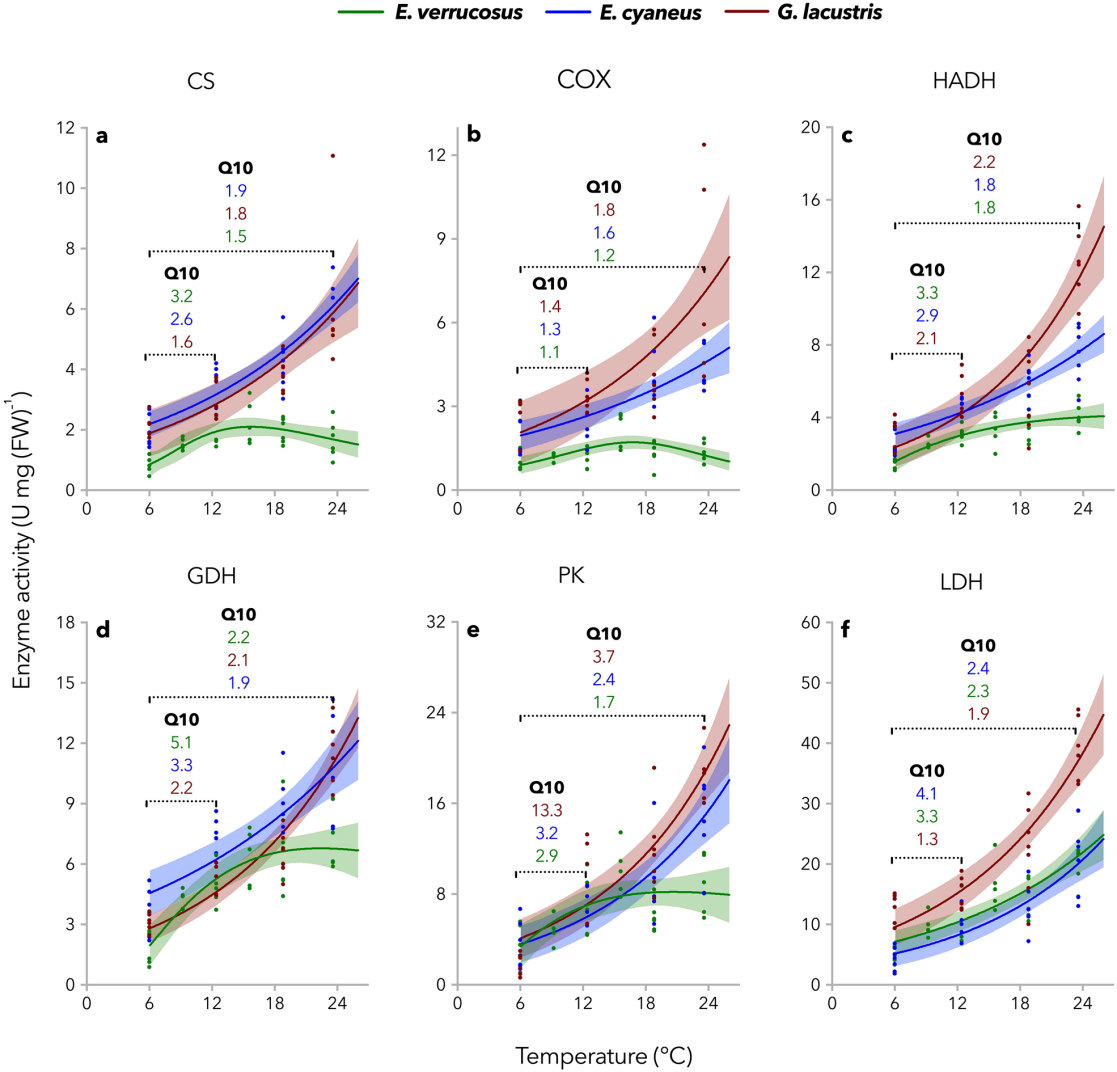
Species-specific thermal reaction norms of maximum enzyme activities. Citrate synthase (CS), cytochrome-c-oxidase (COX), 3-hydroxyacyl-CoA dehydrogenase (HADH), glutamate dehydrogenase (GDH), pyruvate kinase (PK), and lactate dehydrogenase (LDH) were extracted from tissues of *E. verrucosus*, *E. cyaneus* and *G. lacustris* exposed to gradual temperature increase (0.8 °C d^−1^). Samples of all three species were taken at 6 °C, 12.4 °C, 18.8 °C, and 23.6 °C; besides, samples of *E. verrucosus* were taken at 9.2 °C and 15.6 °C. Measurements were performed at the respective sampling temperatures. Enzyme activities level off at temperatures exceeding about 15 °C in the stenothermal Lake Baikal endemic amphipod species *E. verrucosus* (except for LDH). At the same time, they increase exponentially over the entire thermal range in the two more eurythermal species *E. cyaneus* (Lake Baikal, endemic) and *G. lacustris* (Holarctic, ubiquitous). Dots represent raw data. Solid lines represent nonlinear models (equations are summarized in Table S2; Supplement), and shaded areas display 95% confidence intervals (n = 5–7).

### Quantification of transcript levels

The assembled transcriptomes of *E. verrucosus*, *E. cyaneus* and *G. lacustris* were used to derive the coding sequences of the genes of interest^29^. In brief, mRNA from whole individuals was extracted, and cDNA was sequenced on an Illumina HiSeq2000 (Illumina, San Diego, USA). The trimmed 101 bp paired-end reads were assembled using Trinity^30^. A small database with the sequences from the closest taxa available was set up for each gene and used as a query against the transcriptome assemblies using tblastx (NCBI). The best hits for each gene were reassembled with CAP3^31^. Spurious gene assemblies were removed after validation against the complete NCBI database with BLAST. Eventually, the most reliable contig was used for primer design and quantification (see Table 1 for accession numbers).

**Table 1:** Genes and primer sequences for real-time PCR. The primer pairs were derived from the genes of interest from the respective species and used after validation (see material and methods) for RT-PCR for the respective organism: *E. verrucosus* (Ev), *E. cyaneus* (Ec), and *G. lacustris* (Gl).

Organism	Gene ID	Annotation (Swissprot)	Accession No	Forward-Primer	Backward primer	Used for:
*E. verrucosus*	CISY	Citrate synthase	KX831868	CACCTCCCTGATGACAACCTTT	GCCGGGCACCACAGTATAA	Ev, Ec
				GGAGTGCCAACTTCACCAACA	GCCTCATGAGCTCGGTAAACTG	Gl
	COX4	Cytochrome c oxidase subunit IV	KX831869	GAAAAGGAAAAGGGTGACTGGAA	AGATGCTCGGTATAAGGCCTTCT	Ev, Ec, Gl
	COX2	Cytochrome c oxidase subunit II	YP_008964129	GTAGCCCTACCAATTAACACTCAAATT	CCGCCCATGAGTGAATAACG	Ev
	GLUD1	Glutamate dehydrogenase 1, mitochondrial	KX831872	GTGGTGTCACCGTTTCCTACTTT	GGTGAGCCTGCCGTAAGAGA	Ev, Ec, Gl
	ECHA	Trifunctional enzyme subunit alpha, mitochondrial, includes: 3-hydroxyacyl-CoA dehydrogenase	KX831870	AAGTGGTGATTGTGGTGAAGGA	AACATGGGTGCCAGGATACG	Ev, Ec
	LDH-A	Lactate dehydrogenase A chain, muscle isoform	KX831873	TCGGCCTGTCCGTAGCA	GCAACGTCTCTGGTTCTTCATG	Ev, Ec
	PKM	Pyruvate kinase, muscle isoform	KX831875	CGCCATCATCGTCATTACCA	AACGAGGCCGGTACTTGGA	Ev
*E. cyaneus*	ACT	Actin	JN860427	CCGCCGAGCGAGAAATC	GGGCCACGTAGCAAAGCTT	Ec, Ev, Gl
	G3P	Glyceraldehyde-3-phosphate dehydrogenase	KF293381	GGCAAAGGTCCACTTCAACAA	GGCGGAGGGAGCAGAGAT	Ec, Ev, Gl
	PKM	Pyruvate kinase, muscle isoform	KX844831	CTGCGGTGGAGGCTTCTTT	GTGGTCGTGATGACGATGATG	Ec
*G. lacustris*	ECHA	Trifunctional enzyme subunit alpha, mitochondrial, includes: 3-hydroxyacyl-CoA dehydrogenase	KX831871	GGCCTATGGAGCAGAGTTTGA	TTCCAGATTTGGCGTAGTCTTG	Gl
	LDH-A	Lactate dehydrogenase A chain, muscle isoform	KX831874	GCCTGCGCGACCTCAA	CGTTGTACTTGTCAGGGTCATTG	Gl
	PKM	Pyruvate kinase, muscle isoform	KX831876	CTGCCGCAACATAGATTCCA	TCACCGCGAGCGATCAT	Gl

To quantify transcript levels, total RNA was isolated from pooled animals by a combined phenol and column-based protocol. Briefly, 40–60 mg tissue was homogenized using a Precellys 24 system (Bertin Technologies, USA) in 1 ml of Qiazol reagent according to the manufacturer (Qiagen, Hilden, Germany) until phase separation. Consecutively, the water phase containing the RNA was cleaned using the clean-up procedure of the RNeasy kit (Qiagen) according to the manufacturer’s protocol. RNA quantity, quality and integrity were examined as described earlier^33^. cDNA was synthesized using the High-Capacity cDNA Reverse Transcription kit (Applied Biosystems, USA after removing DNA contaminations with the Turbo DNA-free kit (Ambion, USA)^34^. Quantitative real-time PCR (qRT-PCR) of 2 ng cDNA was performed on a ViiA™ 7 Real-Time PCR System (Applied Biosystems, USA) with SYBR Green PCR master mix (Applied Biosystems, USA) and 300 nM of each primer. Primers were designed using PrimerExpress (version 3.0, Applied Biosystems, Darmstadt, Germany) (Table 1). All primers were tested for all three species over five serial cDNA dilutions (2 ng to 0.02 pg); the efficiency (E) was 2.0 ± 0.1 and R_2_ > 0.98. After each qRT-PCR run, melting curves were evaluated to corroborate the specificity of the primer products^34^.

To identify suitable endogenous controls and valuable interspecies comparisons, the most stable genes within a larger set of candidate genes were identified through NormFinder (version 0.953) (MOMA, Aarhus University Hospital, Denmark). *Actin* and *gapdh* transcript levels were similarly stable in all three species under the experimental conditions and used for normalization. Expression of each gene was determined as log2fold change (log2FC) of individual expressions of treatment and control samples normalized to the control’s mean.

#### Overall expression of ribosomal protein genes

RNA sequencing was described in detail earlier^29^. The relative abundance of each transcript (log2fold change compared to the initial control) was estimated with salmon^35^ and DESeq2^36^ packages for the R statistical environment (R Core Team, 2017) using scripts provided by Trinity^37^, described earlier^38^. Expression of the transcripts encoding ribosomal proteins was summarized with custom R-scripts (https://github.com/drozdovapb/EveEcyGlaDE/tree/master/gradual_temperature_increase).

The raw and processed data are available at NCBI. (https://www.ncbi.nlm.nih.gov/geo/query/acc.cgi?acc=GSE129069).

### Data analysis

Enzyme activities were measured in freshly prepared tissue extracts at the respective sampling temperature, as this resembles best in-situ conditions. These measurements served to obtain an ecologically relevant picture of physiological processes and to enable to integrate data with whole animal responses that were previously determined along the same thermal gradient. Further, maximum enzyme activities were determined at common assay temperatures of 6 °C and 18.8 °C to study the thermal sensitivity of the enzymes in dependence of sampling temperature. To examine the effect of measuring temperature on enzyme activity, we determined enzyme activities of samples taken at 12.4 °C also at 6 °C, 18.8 °C, and 23.6 °C. As described for whole animal responses such as oxygen consumption and ventilation, data of maximum enzyme activities were fitted to nonlinear models, preselecting candidate models by shape using the Dynamic Fit Wizard in SigmaPlot (version 13, Systat Software Inc., USA/Canada) that provides a selection of 120 different models; the Akaike’s information criterion (AICc) was used to define the best-fit models and those with the lowest AICc was chosen^12^. Curve fitting was used to visualize the different thermal reaction norms of key metabolic enzymes in the three studied amphipod species. Further, it served as a geometric tool to determine the points where the curves start to flatten out and approach zero. According to the nonlinear regression approach of Marshall et al.^39^, this breakpoint temperature (BPT) is reached when the model’s slope equals 0.065. This approach proposes to fit various curvilinear functions to continuous physiological data instead of using broken stick regression, as nonlinear regression approaches were more accurate and provided more powerful hypothesis tests.

Changes in transcript levels were identified by one-way ANOVA and the subsequently performed Student–Newman–Keuls post hoc test (α < 0.05; SigmaPlot version 12, Systat Software Inc., USA/Canada).

## Results

### Maximum enzyme activities at in-situ temperatures

Enzyme activities determined in the two Baikal endemic amphipod species *E. verrucosus* (stenothermal) and *E. cyaneus* (eurythermal) and in the closely-related widespread Holarctic amphipod *G. lacustris* (eurythermal) showed species-specific patterns. While activities of all investigated enzymes increased exponentially over the entire thermal range (6–23.6 °C) in *E. cyaneus* and *G. lacustris* in line with common Q_10_-behavior, they leveled off in *E. verrucosus* at temperatures exceeding about 15 °C (Fig. 2). For *E. verrucosus*, Q_10_-values exceeding the value of ~ 2–3 (typical for most biological systems) were found for most enzymes in the first half of the thermal trial (6–15.6 °C), which majorly accounts for the peculiar curve shapes determined for this species. Due to these patterns, enzyme activities in the second half of the experimental exposure were generally substantially higher in *E. cyaneus* and *G. lacustris* than in *E. verrucosus*. If not otherwise indicated, enzyme activities are given as means ± s.e.m. For example, for CS at 23.6 °C, activities of 1.68 ± 0.25, 6.23 ± 0.29, and 6.14 ± 1.00 U per mg FW were measured in *E. verrucosus*, *E. cyaneus*, and *G. lacustris*, respectively (Fig. 2a). Only LDH activity (indicator of anaerobic glycolytic capacity) increased exponentially in *E. verrucosus* over the entire investigated thermal range, and activities in the two Baikal species were very similar (*E. verrucosus* : 21.28 ± 1.92 and *E. cyaneus*: 20.64 ± 2.43 U per mg FW at 23.6 °C; Fig. 2f). Instead, LDH activities were higher in *G. lacustris* than in both *E. cyaneus* and *E. verrucosus*, especially at higher temperatures, indicating higher anaerobic glycolytic capacities in the Holarctic species in the warmth (*G. lacustris*: 39.16 ± 2.14 U per mg FW at 23.6 °C; Fig. 2f).

In *E. verrucosus*, BPTs of 13.5 °C and 17.1 °C were determined for CS and COX, respectively (Fig. 2a,b). Comparing the two eurythermal species, COX activities were higher in *G. lacustris* than in *E. cyaneus* at 23.6 °C (*G. lacustris*: 7.56 ± 1.37 and *E. cyaneus*: 4.28 ± 1.37 U per mg FW; Fig. 2b). At 18.8 °C and 23.6 °C, also HADH activities (indicator for fatty acid metabolic activity) were higher in *G. lacustris* than in *E. cyaneus;* the modelled curves show no overlap of the 95% confidence intervals (Fig. 2c). Mean HADH activities were 4.01 ± 0.29, 7.46 ± 0.59, and 12.63 ± 0.84 U per mg FW at 23.6 °C for *E. verrucosus*, *E. cyaneus* and *G. lacustris*, respectively; Fig. 2c). Especially low PK activities were measured for *G. lacustris* at 6 °C that deviated from the derived exponential curve (Fig. 2e). Moreover, PK activities of *G. lacustris* at 6 °C were low compared to the two Baikal endemic amphipod species (*G. lacustris*: 1.84 ± 0.39, *E. cyaneus*: 3.36 ± 0.78, and *E. verrucosus*: 3.61 ± 0.84 U per mg FW; Fig. 2e). However, except for PK activities being lower at 6 °C in *G. lacustris* compared to the two Baikal species, all three species showed similar PK activities up to 15.6 °C. At low temperature (6 °C), GDH activities (representing a key link between catabolic and anabolic pathways) were substantially lower in the stenothermal *E. verrucosus* (1.92 ± 0.84 U per mg FW) in comparison to its eurythermal sister species *E. cyaneus* (3.51 ± 0.44 U per mg FW; Fig. 2d).

To verify that incubation (sampling) temperature and not simply assay (measuring) temperature was the reason for the peak and hyperbolic curves of functional capacities derived for *E. verrucosus* (Fig. 2), samples that were taken at 12.4 °C were analyzed at 6 °C, 12.4 °C, 18.8 °C and 23.6 °C, which resulted in exponential curves for all species, including *E. verrucosus* (Fig. S1; Supplement). Further, to exclude that the factor time was responsible for the species-specific patterns of enzyme activities, results for the time control (kept at 6 °C throughout the experimental exposure) are shown in Fig. S2 (Supplement) along with samples taken from the thermal trial (all samples were determined at an assay temperature of 18.8 °C). Here, controls remained stable, reflected by horizontal lines. Instead, enzyme activities determined in *E. verrucosus* samples from the thermal increase exposure showed bell-shaped curves and for *G. lacustris* slightly U-shaped curves. The curves derived for *E. cyaneus* did not deviate from controls.

Enzyme activities of samples taken along the thermal trial were determined at 6 °C and 18.8 °C to derive Q_10_-values and gain information about the enzymes’ thermal sensitivity in dependence of the ambient temperature. The analysis (ANOVA and subsequent Holm-Sidak test; p < 0.05) revealed that COX and PK were especially thermally sensitive at 12.4 °C, as significantly higher Q_10_-values of COX were measured in *E. verrucosus* samples taken at 12.4 °C compared to those of samples taken at all other temperatures except for 15.6 °C (ANOVA (5, 26) = 6.47; p < 0.001). The same was seen for PK, as the Q_10_-values of samples taken at 12.4 °C varied significantly from those taken at 6 °C and 18.8 °C (ANOVA (5, 26) = 5.97; p < 0.001); Table S3; Supplement).

### Shifts in metabolic fuel use

**Figure 3:**
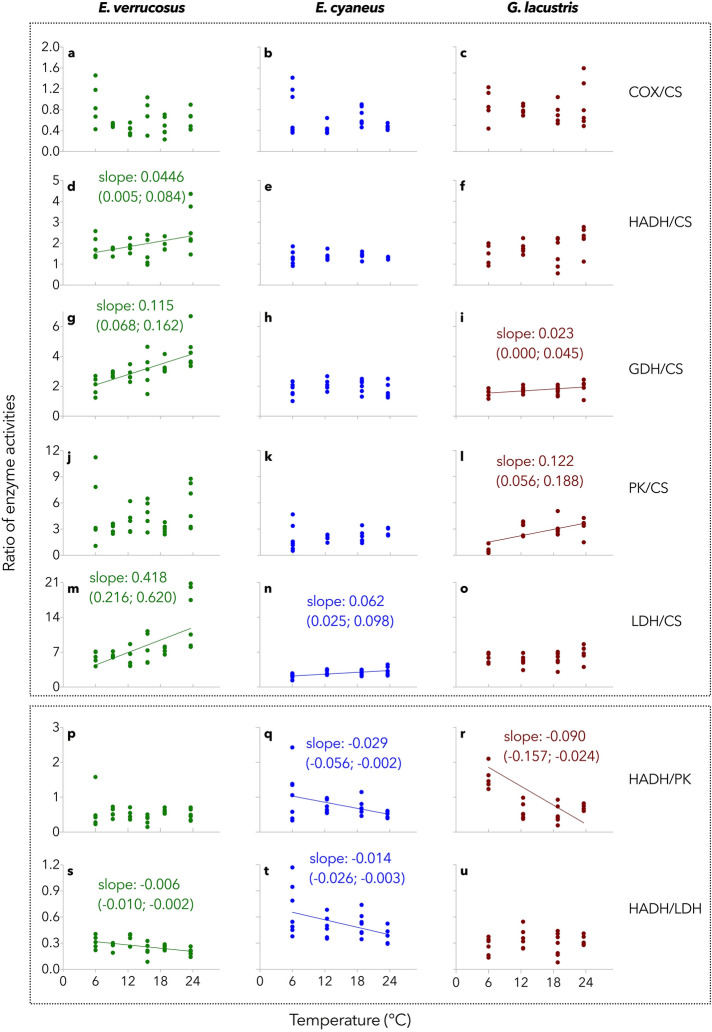
Ratios of maximum enzyme activities. Citrate synthase (CS), as the central hub in metabolism, connecting carbohydrates, lipids, and proteins, is used as denominator to decipher shifts in the use of metabolic fuels. Cytochrome-c-oxidase (COX) is a marker for aerobic capacity, pyruvate kinase (PK) and lactate dehydrogenase (LDH) are used as indicators of anaerobic ATP generation, 3-hydroxyacyl-CoA dehydrogenase (HADH) is essential for fatty acid metabolic processes, and glutamate dehydrogenase (GDH) represents a key link between catabolic and anabolic protein metabolism. A positive slope indicates a relatively increasing contribution of the respective enzyme (enzyme in the numerator position) to energy metabolism along with warming. Oppositely, a negative slope indicates a decreasing contribution of the enzyme in the numerator position to metabolic processes. For a comparison of fatty acid-based metabolism and glycolysis, HADH activity was related to PK and LDH activity. Measurements of enzyme activities in the tissue extracts of *E. verrucosus*, *E. cyaneus*, and *G. lacustris* were performed at the respective sampling temperatures. Individuals of all species were exposed to a gradual temperature increase (0.8 °C d^−1^). Slopes and their 95% confidence intervals are indicated when simple linear regression analysis revealed that slopes were significantly different from zero (p < 0.05). Dots represent ratios of individual animals (one data point, i.e., HADH/PK ratio with the value of 6 in the data set *G. lacustris—*6 °C, is not depicted to avoid reducing the resolution of the panels but was not removed from any analysis); n = 4–7.

Citrate synthase, as the key enzyme of the tricarboxylic acid (TCA) cycle, represents the central hub in metabolism, connecting carbohydrates, lipids, and proteins. Therefore, changed ratios between key enzymes of the respective pathways and CS may indicate metabolic shifts upon the warming treatment. With rising temperature, a progressive shift to a higher disposition for anaerobic glycolytic capacity than aerobic capacity was observed for *E. verrucosus* and *E. cyaneus* indicated by progressively increasing LDH/CS ratios along with the experimental temperature increase (Fig. 3m,n). Here, simple linear regression analysis revealed that slopes were significantly different from zero (p < 0.05). However, LDH/CS ratios increased only marginally in *E. cyaneus* (slope: 0.062; p = 0.0018), but considerably in *E. verrucosus* (slope: 0.418; p = 0.0002). With respect to the aerobic capacity, the protein catabolic capacity increased with rising temperatures indicated by increasing GDH/CS ratios for *E. verrucosus* (slope: 0.115; p < 0.0001), but not in *E. cyaneus* and only slightly in *G. lacustris* (slope: 0.023; p = 0.0458, Fig. 3g–i).

The ratios of capacities in fatty acid to carbohydrate metabolism indicated by HADH/PK and HADH/LDH capacities are depicted in the lower box of Fig. 3. In *E. verrucosus*, no changes in the HADH/PK ratios were detected; only HADH/LDH ratios decreased with increasing temperature (slope: -0.006; p = 0.0032, Fig. 3p,s). In *E. cyaneus*, both HADH/PK and HADH/LDH ratios decreased slightly with increasing temperature (slopes: -0.029 (p = 0.0336) and -0.014 (p = 0.0186), respectively; Fig. 3q,t). HADH/PK decreased with increasing temperature in *G. lacustris* (slope: − 0.090; p = 0.0099), but the HADH/LDH ratio remained stable (Fig. 3r,u).

### Changes in mRNA transcript levels

**Figure 4:**
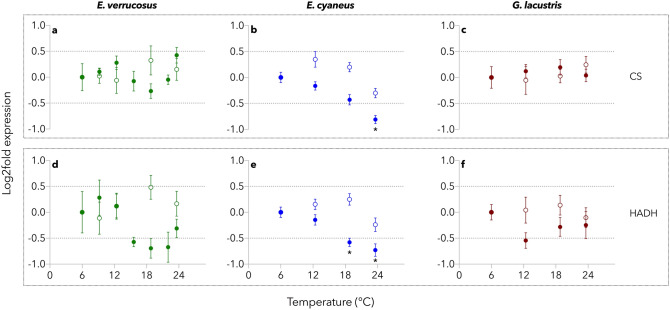
Temperature-dependent expression of RNA transcripts of key metabolic enzymes. Transcripts of citrate synthase (CS) and 3-hydroxyacyl-CoA dehydrogenase (HADH) were analyzed in whole animal extracts of *E. verrucosus*, *E. cyaneus*, and *G. lacustris* upon exposure of the animals to gradual temperature increase (0.8 °C d^−1^). Samples of all three species were taken at 6 °C, 12.4 °C, 18.8 °C, and 23.6 °C; besides, samples of *E. verrucosus* were taken at 9.2 °C, 15.6 °C, and 22 °C. Expression data are expressed as log2fold change calculated against the same endogenous controls (*actin* and *gapdh*) for all three species. Temperature-treated groups are shown with filled circles, whereas the time control groups (kept at 6 °C) are represented by open circles. Asterisks indicate data points outside the range of biological insignificance (± 0.5 log2fold change; dotted horizontal lines), which are significantly different from the 6 °C treatment group (ANOVA, p < 0.05). Data are presented as means ± s.e.m. (n = 5–7).

The main pattern of temperature-dependent changes in RNA transcript levels determined by qRT-PCR of the studied enzymes is shown exemplarily for CS and HADH (Fig. 4). The panels for COX, GDH, PK, and LDH are shown in Fig. S3 (Supplement). In the time control at 6 °C, RNA transcript levels of the studied enzymes varied to a certain degree in all species but did not show any significant alterations or trends over time (Fig. 4 and Fig. S3 (Supplement), open circles). Against this background, statistical results of the treatment groups were only considered biologically significant if the log2fold change was greater than ± 0.5.

RNA transcript levels, except for PK (Fig. S3h; Supplement), decreased progressively in *E. cyaneus* with the gradual warming (Fig. 4b,e and Fig. S3b,e,k; Supplement). In *E. verrucosus*, expression of most genes fluctuated around the control levels (Fig. 4a,d and Fig. S3a,d,g,j; Supplement). In *G. lacustris*, mostly stable RNA transcript levels or enzyme-specific trends were observed along with the warming treatment (Fig. 4c,f and Fig. S3c,f,i,l; Supplement). Only LDH decreased significantly at 12.4 °C and 18.8 °C but turned back to control levels at the end of the trial (ANOVA (3, 20) = 5.524; p = 0.0063), (Fig. S3l; Supplement).

A cluster of ribosomal transcripts was investigated through RNA sequencing. In *E. verrucosus*, the expression of about 300 ribosomal transcripts was decreased by up to ~14.7-fold only at the highest temperature of the thermal trial (24.4 °C). Instead, the expression remained unchanged in *E. cyaneus* during the entire thermal trial and increased slightly in *G. lacustris* at the highest temperature (Fig. S4).

## Discussion

In this study, the metabolic background of whole animal responses to gradually increasing temperature of three amphipod species, *E. verrucosus*, *E. cyaneus*, and *G. lacustris*, was investigated. We aimed to assess whether key enzymes of energy metabolism in the stenothermal *E. verrucosus* are specifically adapted to the narrow thermal window previously determined at the whole animal level. Further, we aimed to reveal whether *E. cyaneus* (endemic to Lake Baikal; eurythermal) and *G. lacustris* (ubiquitous in the Holarctic; eurythermal) show species-specific metabolic alterations despite their similar thermal tolerance and analyzed how metabolic fuel use changed with rising temperature.

All investigated key metabolic enzymes except for LDH showed peculiar curve shapes in *E. verrucosus* (endemic to Lake Baikal, stenothermal) as enzyme activities extracted from the pre-exposed animals and measured at the same assay temperatures increased strongly up to about 15 °C, whereupon they leveled off. This pattern could not be explained by changes in gene expression levels of the respective enzymes. In contrast, enzyme activities extracted from *E. cyaneus* and *G. lacustris* increased with temperature over the entire range of thermal exposure, indicating a lack of thermal compensation at the functional levels during the three weeks of exposure. However, generally higher activities were determined in *G. lacustris* than in *E. cyaneus* in the second half of the thermal trial. Gene expression levels revealed uncompensated activities in *G. lacustris* but thermal compensation in *E. cyaneus*, explaining the more gently inclining slopes in *E. cyaneus*. In all species, metabolic fuel use shifted towards glycolytic energy production under the progressive warming scenario. The following discussion aims at connecting these results to the whole animal responses, behaviors, and thermal niches of the three species determined in previous studies.

### *Eulimnogammarus verrucosus*: From activation to limitation of metabolic activity

#### Responses at different organizational levels

**Figure 5:**
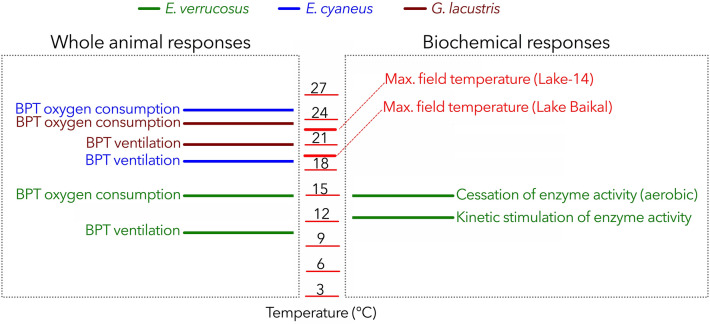
Summary of physiological responses at the whole animal and the biochemical level. Breakpoints (BPTs) indicate limitations/changes of physiological processes in *E. verrucosus* (stenothermal, Lake Baikal endemic), *E. cyaneus* (eurythermal, Lake Baikal endemic), and *G. lacustris* (eurythermal, Holarctic ubiquitous) determined in thermal increase experiments (0.8 °C per day, start temperature: 6 °C). In the two eurythermal species, *E. cyaneus* and *G. lacustris,* no breakpoints of biochemical measures (i.e., capacities of key metabolic enzymes) were determined in the studied thermal range (6–23.6 °C). In the stenothermal *E. verrucosus*, the BPT of routine metabolic rate (measured as oxygen consumption) correlates with the cessation of activities of key metabolic enzymes that rely on molecular oxygen availability.

Routine metabolic rate, analyzed in our previous studies^12^, and activities of key metabolic enzymes determined in the present study leveled off in *E. verrucosus* when a temperature of around 15 °C was exceeded (Fig. 5). Exceeding the congruent BPTs of enzyme capacities and routine metabolic rate resulted in increased mortality of *E. verrucosus,* indicating that a progressive temperature increase beyond 15 °C is critical for *E. verrucosus*^12^. Only LDH enzyme activity, representing the anaerobic capacity of glycolytic metabolism, increased beyond 15 °C in the stenothermal *E. verrucosus*. LDH activity levels and the functional properties of this enzyme reflect the capacity for anaerobic energy generation and consequently, the level of resistance to oxygen deficiency during hypoxia, extensive exercise, or thermal stress^40,41^. Thus, the kinetic activation of LDH indicates an acclimatory response of *E. verrucosus* to systemic hypoxia at higher temperatures and may serve as a short-term ATP-generating system under critical conditions. Accordingly, lactate levels determined during a short-term thermal increase experiment rose at 11 °C and ≥ 15 °C^21^.

The strong activation of enzymatic activity in the first half of the thermal trial is likely due to whole organism feedbacks regulating enzyme activities. Metabolic enzymes have evolved oxygen-sensing mechanisms regulating aerobic and anaerobic energy production^42^. The same features that regulate energy metabolism also regulate transcription of metabolic enzyme genes, so that enzyme activity and transcription are regulated simultaneously, albeit in different time courses and signaling pathways^42^. Moreover, it is important to point out that activation energy is temperature-dependent^43,44^. Thus, in *E. verrucosus*, activation energy varied with sampling temperature in *E. verrucosus*, as indicated by shifts in the thermal sensitivity in dependence of sampling temperature (Table S3; Supplement) and contributes to shaping metabolic performance curves (Fig. 2).

Ventilation showed an earlier onset of limitation than oxygen consumption not only in *E. verrucosus* but also in *E. cyaneus* and *G. lacustris*^12^. We suggested that heart rate needs to largely increase beyond these breakpoints to support further increments in oxygen consumption, as seen in other crustaceans^45^. Such a response can most likely not be sustained in the long term and may constitute an emergency system during short heatwaves. For *E. verrucosus*, the BPT of ventilation was between 10–11 °C. Noticeably, it occurs in parallel with the kinetic stimulation of key metabolic enzymes (Fig. 5). Also, a significant increase of the heat-shock protein Hsp70 was determined at 11 °C and 13 °C in short-term thermal increase experiments, indicating stress at these temperatures^21,46^.

In conclusion, *E. verrucosus* slides from a strong activation at temperatures up to 15 °C into a limitation at the thermal extreme of the studied thermal range (23.6 °C), explaining the thermal reaction norm of aerobic enzymes in *E. verrucosus*. As assay temperature itself did not lead to a similar depression of activity, an active downregulation of the investigated functional capacities beyond 15 °C may be proposed to avoid an undamped development of capacities with warming. At unchanged mRNA levels, post-transcriptional processes including translational efficiency and protein modification may prevail. The observed strong activation of enzymatic activity in the first half of the thermal trial is likely due to whole organism feedbacks regulating enzyme activities. The same mechanism may act beyond 15 °C. However, the major decrease in transcription of ribosomal protein transcripts at 24 °C likely indicates a general decrease in the entire translational machinery at these, for *E. verrucosus*, extreme conditions.

#### Thermal niche and reaction norms of key metabolic enzymes

**Figure 6:**
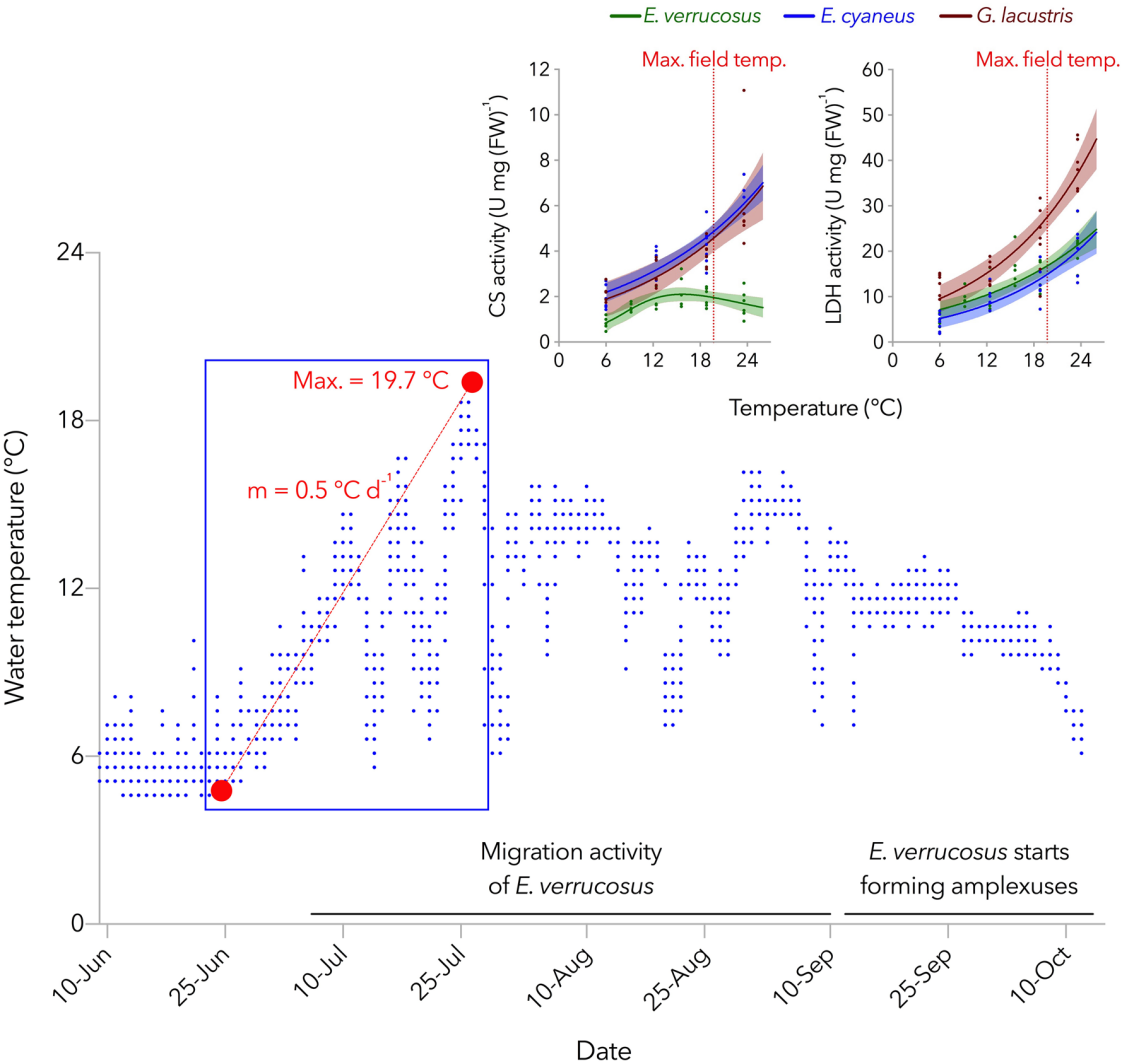
Water temperature in Lake Baikal (Bolshie Koty) at 1 m depth. The temperature was determined with an iButton Temperature Logger (Maxim Integrated, USA) deployed in the benthic zone, every hour from the 9^th^ of June until the 10^th^ of October 2019 (resolution: 0.5 °C). Small panels in the upper right corner show activities of citrate synthase (CS, as indicator of aerobic capacity) and lactate dehydrogenase (LDH, as indicator of anaerobic capacity) measured in *E. verrucosus* (stenothermal, Lake Baikal endemic), *E. cyaneus* (eurythermal, Lake Baikal endemic), and *G. lacustris* (eurythermal, Lake Baikal endemic) along an experimental thermal gradient (0.8 °C per day) in relation to maximum field temperature. Maximum activities of aerobic key metabolic enzymes (e.g., CS) fail to increase steadily with temperature over the entire natural thermal range in the stenothermal *E. verrucosus*, while they increase with temperature over the entire thermal range in *E. cyaneus* and *G. lacustris*. Instead, LDH increases exponentially with temperature in all species over the entire thermal range. Migration of *E. verrucosus* to deeper waters was indicated by decreasing abundances in the upper littoral when temperatures increased in summer.

It was previously observed that *E. verrucosus* is behaviorally adapted to escape unpreferred temperatures as adult individuals migrate from the upper littoral to deeper waters when temperatures rise in summer^11,12^. The congruence of BPTs at the whole animal and biochemical level in *E. verrucosus* is remarkable and possibly due to the close adaptation of this species to its specific thermal niche, necessitating temperature-dependent migration behavior during summer, since a temperature of 15 °C (= Tc of *E. verrucosus*) is in the recorded temperature range in Bolshie Koty bay, and maximum temperatures of 20 °C were measured (Fig. 6). Its large body size and associated competitive strength may allow this specific thermal behavior in the littoral zone, which is inhabited by more than 300 different amphipod species and subspecies^7^. Higher Q_10_-values (6–18.8 °C) were determined in samples taken at 12.4 °C for both COX and PK. Strong kinetic stimulation of enzymatic processes in *E. verrucosus* in this thermal range results in higher activity levels at moderate temperatures, supporting competition, and migration activity of *E. verrucosus* in the field. In September, more or less stable temperatures of around 12 °C prevail in Lake Baikal’s littoral zone in Bolshie Koty, and animals start to form amplexuses (Fig. 6); actual reproduction occurs at water temperatures of 1.5–6 °C^47^. Again, the kinetic stimulation of key metabolic enzymes may support the formation of precopulas and finally, the mating success. Preference temperatures of 5–6 °C were determined in behavioral studies with adult individuals of *E. verrucosus*^17,48^. In this thermal range (e.g., in the 6 °C controls tanks), *E. verrucosus* only occasionally showed swimming activity. *Eulimnogammarus verrucosus* represents the middle littoral and sublittoral fauna of amphipods of Lake Baikal’s rocky beaches, where it dominates by number and biomass^10^. This faunistic complex comprises many thermally sensitive species, predominantly reproducing in winter, such as *E. verrucosus*^49^. The peculiar curve shapes of temperature-dependent enzyme activities are likely due to the high need for specialization to a certain (thermal) niche in this hotspot of amphipod biodiversity.

In summary, the universal curve shapes of maximum enzyme activities in *E. verrucosus* characterized by large Q_10_-values up to about 15 °C and then sudden cessation of activities allow higher metabolic activation at lower temperatures compared to the sister species. This beneficial kinetic trait at low temperature supporting locomotor activity, mating behavior, and thus competitiveness at the preferred habitat temperature, is compensated for by avoiding high temperatures through migration to colder waters during summer.

### *Eulimnogammarus cyaneus* and *Gammarus lacustris*: same same (thermal tolerance) but different (metabolic phenotype)

#### Responses at different organizational levels

Despite the similarities in whole organism thermal tolerance in *G. lacustris* and *E. cyaneus* indicated by similar BPTs for oxygen consumption (22.9–25.2 °C for *E. cyaneus* and about 23.6 °C for *G. lacustris*) and ventilation (19.1–20.8 °C for *E. cyaneus* and 21.1–21.8 °C for *G. lacustris*)^12^ (Fig. 5), clear differences in metabolic thermal responses were found in this study. The findings of uncompensated metabolic activities as well as higher capacities of mitochondria (i.e., COX), fatty acid metabolism and anaerobic glycolysis in *G. lacustris* than in *E. cyaneus* indicate differences in temperature-dependent movement activity. First, species with high locomotor activity rates may show increased cristae surface areas per unit of mitochondrial volume and consequently high COX/CS ratios^50^. Second, the high need for sustained swimming was shown to be indicated by high aerobic capacities and efficient energy production through fatty acid metabolism^51^. Third, species with a high need for immediate movement responses, e.g., needed for escaping predators, were shown to have high anaerobic capacities^52^. As the three overarching tasks, to find food, escape predators, and reproduce, are controlled by associated encounter rates, natural selection will favor the most beneficial locomotor activity^53^. The observed differences in the two eurythermal amphipod species’ metabolic performance may thus be explained by specific habitat features and different lifestyles.

#### Habitat and metabolic performance

Less steep increases of enzyme activities in *E. cyaneus* compared to *G. lacustris* and reduced expression levels of most investigated transcripts indicate some thermal compensation in *E. cyaneus*. This thermal compensation may be explained by the trophic state of the habitat. *E. cyaneus* is clearly restricted to the upper littoral of Lake Baikal due to the high competition between amphipod species inhabiting the littoral zone^7^, and some level of thermal compensation resulting in a smaller seasonal amplitude of metabolic activity compared to *G. lacustris* might be beneficial in this oligotrophic habitat as a high metabolic activity comes along with increased fuel usage. Instead, *G. lacustris* living in more eutrophic waters might benefit from highly increased metabolic rates during the short summer season as this goes along with high movement activity. This optimized performance at warm temperatures is supported by a higher efficiency of the translation machinery and uncompensated expression of the investigated transcripts.

The large anaerobic capacity of *G. lacustris* compared to that of the Baikal amphipods determined in this study goes along with adaption to hypoxic conditions. Some habitats of *G. lacustris*, like small ponds, are entirely frozen in Siberian winters and consequently hypoxic. During summer, these eutrophic habitats may become hypoxic due to the high bacterial turnover of organic matter. Instead, Lake Baikal water is constantly oxygen-saturated up to a depth of 500 m^54^. Thus, Baikal endemic amphipods may not need such a strong anaerobic backup system and, noticeably, LDH activities in Baikal amphipods were similar during the entire thermal trial.

#### Lifestyle and metabolic performance

The much higher anaerobic glycolytic capacity of *G. lacustris* compared to both Baikal species may be needed for escaping predators by tail flipping, which provides the highest speeds and acceleration, similar to burst swimming in fishes^51^. It is supported by anaerobic energy production, as shown for shrimps and other crustaceans^55,56^. Large immature and adult individuals of *G. lacustris* are cannibalistic^57^. Thus, a fast escape response increases the intraspecific fitness of *G. lacustris*. There is likely a strong selection for high anaerobic capacity in *G. lacustris* as the life-dinner principle predicts a stronger selection for running for survival than running for food^58,59^. By contrast, no cannibalism was seen in *E. cyaneus.*

*Eulimnogammarus cyaneus* mainly inhabits the upper littoral close to the water edge throughout the year and represents a summer-reproducing complex of Baikal amphipods ^6^. *Gammarus lacustris* inhabits shallow-water habitats across the Holarctic and is thus exposed to highly variable thermal conditions; like *E. cyaneus*, it mainly reproduces in summer but has a much more versatile reproductive activity^57^. To support reproductive activity in summer, which is promoted by higher locomotor activity, both species need to refill energy stores. However, the depletion of energy stores (and associated high need for movement activity) after the long winter season is likely much higher for *G. lacustris*, as this species shows highly decreased activity in winter. Accordingly, large amounts of adult *G. lacustris* were found inactive in leaf litter^12^. Further, reproductive success is determined by the encounter rate of males and females, and lower population densities of *G. lacustris* than those of *E. cyaneus* require high cruising activity and consequently, as described above, efficient ATP production fostered by high mitochondrial capacities and fatty acid metabolism.

### Shifts in metabolic fuel use

The rising capacity of anaerobic glycolysis with increasing temperature in *E. verrucosus* likely indicates a higher disposition to meet hypoxemic conditions, which are more frequent at elevated temperatures, where oxygen (capacity) becomes limiting^14,60^. Moreover, the ratio between HADH activity, representing β-oxidation of fatty acids, and LDH activity decreased, indicating a preference for burning carbohydrates in the warmth. Similarly, a shift from lipid-based to carbohydrate-based metabolism in Antarctic fish (*Pachycara brachycephalum*) upon warming was interpreted as ‘warm-hardiness’, as carbohydrates can be metabolized more easily under limited systemic oxygen levels in the warmth^33^. In *E. cyaneus*, the LDH/CS activity ratio increased only slightly, but again a clear shift from HADH to LDH became visible, indicating a similar metabolic shift to the glycolytic pathway and away from β-oxidation with increasing temperatures. *Gammarus lacustris* showed the highest LDH/CS activity ratios at the highest incubation temperature, also indicating a higher involvement of the glycolytic pathway and the highest hypoxia tolerance. A high glycolytic capacity can be mobilized short-term and may be an adaptive trait supporting sudden energy demand^61^. Elevated temperature and high locomotor activity may thus favor the use of the glycolytic metabolic pathway, as energy is readily available and (initially) independent from oxygen supply, whereas fatty acid oxidation relies on the availability of molecular oxygen. Besides, increased GDH/CS activity ratios with rising temperature in *E. verrucosus* indicate protein catabolism involvement under progressive warming.

## Conclusions

This study differentiated the thermal responsiveness and reaction norms of three amphipod species exposed to a seasonal (summer) warming scenario close to the present environmental conditions. The stenothermal Baikal endemic amphipod *E. verrucosus* showed fine-tuned key metabolic enzyme activities in parallel with routine metabolic rates characterized by strong activation of enzymatic activity and sudden cessation at about 15 °C. High thermal sensitivity and activity of key metabolic enzymes at about 12 °C may support locomotor activity to escape deleterious temperatures in the upper littoral by migrating to deeper waters. Accordingly, previous field observations showed decreased abundances of *E. verrucosus* when temperatures in the upper littoral of Lake Baikal rose in summer. Furthermore, the formation of amplexuses requiring cruising activity to increase the encounter rates of potential mates starts in September when relatively stable temperatures of about 12 °C prevail in Lake Baikal’s upper littoral. Similar thermal limits and high thermal plasticity indicated by exponential increases of enzyme activities within the entire studied thermal range (6–23.6 °C) were determined in the two eurythermal species *E. cyaneus* and *G. lacustris*. Nevertheless, striking differences in metabolic profiles were recorded indicating the need for higher locomotor activity with increasing temperature in the widespread Holarctic amphipod *G. lacustris* than in the Baikal endemic *E. cyaneus*. Both anaerobic capacities needed for escape responses and aerobic capacities needed for sustained swimming were higher in the Holarctic species, in accordance with the habitat and lifestyle features discussed above. A progressive shift to glycolytic energy production with rising temperatures was observed in all species, likely supporting faster energy production in the warmth. This was most striking for the cold-stenothermal *E. verrucosus*, where LDH was the only enzyme following the same Q_10_-pattern as in the more eurythermal species. Thus, evolutionary constraints may exist to preserve thermal plasticity for the anaerobic glycolytic pathway in cold-adapted species.

This study shows that, rather than investigating enzyme stabilities, the different regulation patterns of key metabolic enzymes in pre-exposed animals can provide a deeper understanding of the thermal performance as the here presented metabolic profiles reflect the distinct thermal behavior, activity pattern, and finally the temperature-dependent spatial distribution of the studied amphipods within their natural habitats. Consequently, climate change, which majorly alters the thermal regime of Lake Baikal’s littoral, will certainly affect the spatial distribution or Darwinian fitness of the stenothermal *E. verrucosus* due to its close adaptation to its thermal niche. The switch from fatty acid catabolism to glycolytic energy production may be a common mechanism in response to limited oxygen rather than to temperature itself. As fatty acid stores provide a higher calorific value than carbohydrates, the ecological implications of the observed shift in metabolic fuel use need further investigation in the light of climate change.

## Supplementary Information

Supplementary Information.

## Data Availability

Data will be available at the database PANGAEA.
