# Supplementary material for: Thermal reaction norms of key metabolic enzymes reflect divergent physiological and behavioral adaptations of closely related amphipod species
Source: Sci Rep. 2021 Feb 25;11:4562. doi: 10.1038/s41598-021-83748-2 (PMC7907238; doi:10.1038/s41598-021-83748-2)
Supplement: Supplementary file 1 — Supplementary Information. [file 41598_2021_83748_MOESM1_ESM.pdf]

Thermal reaction norms of key metabolic enzymes reflect divergent physiological and behavioral adaptations of closely related amphipod species

Lena Jakob[*] [1], Kseniya P. Vereshchagina [2], Anette Tillmann [1], Lorena Rivarola-Duarte [3], Denis V. Axenov-Gribanov [2], Daria S. Bedulina [2], Anton N. Gurkov [2], Polina Drozdova [2], Maxim A. Timofeyev [2], Peter F. Stadler [3,5,6,7,8,9,10], Till Luckenbach [4], Hans-Otto Pörtner [1], Franz J. Sartoris [1] and Magnus Lucassen [1]

[1] Department of Integrative Ecophysiology, Alfred Wegener Institute Helmholtz Centre for Polar and Marine Research, Am Handelshafen 12, 27570, Bremerhaven, Germany

[2] Institute of Biology, Irkutsk State University, Karl Marx str.1, 664003 Irkutsk, Russia

[3] Bioinformatics Group, Department of Computer Science, University Leipzig, Leipzig, Germany

[4] Department of Bioanalytical Ecotoxicology, UFZ – Helmholtz Centre for Environmental Research, Permoserstr. 15, 04318 Leipzig, Germany

[5] LIFE, Leipzig Research Center for Civilization Diseases, University Leipzig, Leipzig, Germany

[6] Interdisciplinary Center for Bioinformatics, University Leipzig, Härtelstraße 16-18, 04107 Leipzig, Germany

[7] Max Planck Institute for Mathematics in the Sciences, Leipzig, Germany

[8] Department of Theoretical Chemistry, University of Vienna, Wien, Austria

[9] Facultad de Ciencias at Universidad National de Colombia, Bogota, Colombia

[10] Santa Fe Institute, Santa Fe, New Mexico

[*]Corresponding author: Lena.Jakob@awi.de

Supplement

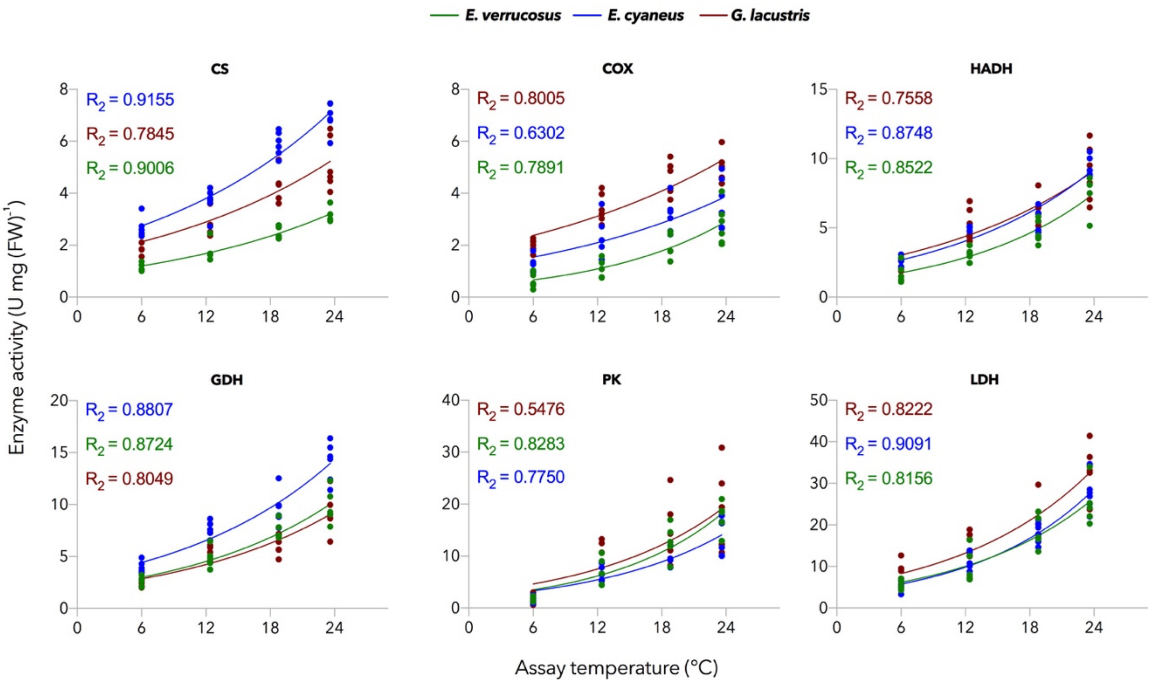

Figure S1: Activities of citrate synthase (CS), cytochrome-c-oxidase (COX), 3-hydroxyacyl-CoA dehydrogenase (HADH), glutamate dehydrogenase (GDH), pyruvate kinase (PK), and lactate dehydrogenase (LDH) in tissue extracts of *E. verrucosus*, *E. cyaneus* and *G. lacustris* sampled at 12.4 °C after increasing temperature from 6 °C to 12.4 °C with a rate of 0.8 °C per day in dependence of assay temperature. Samples were measured at 6 °C, 12.4 °C, 18.8 °C and 23.6 °C. Dots represent raw data and lines are exponential curve fits (Y=Y0*exp(k*X)); n = 4-6.

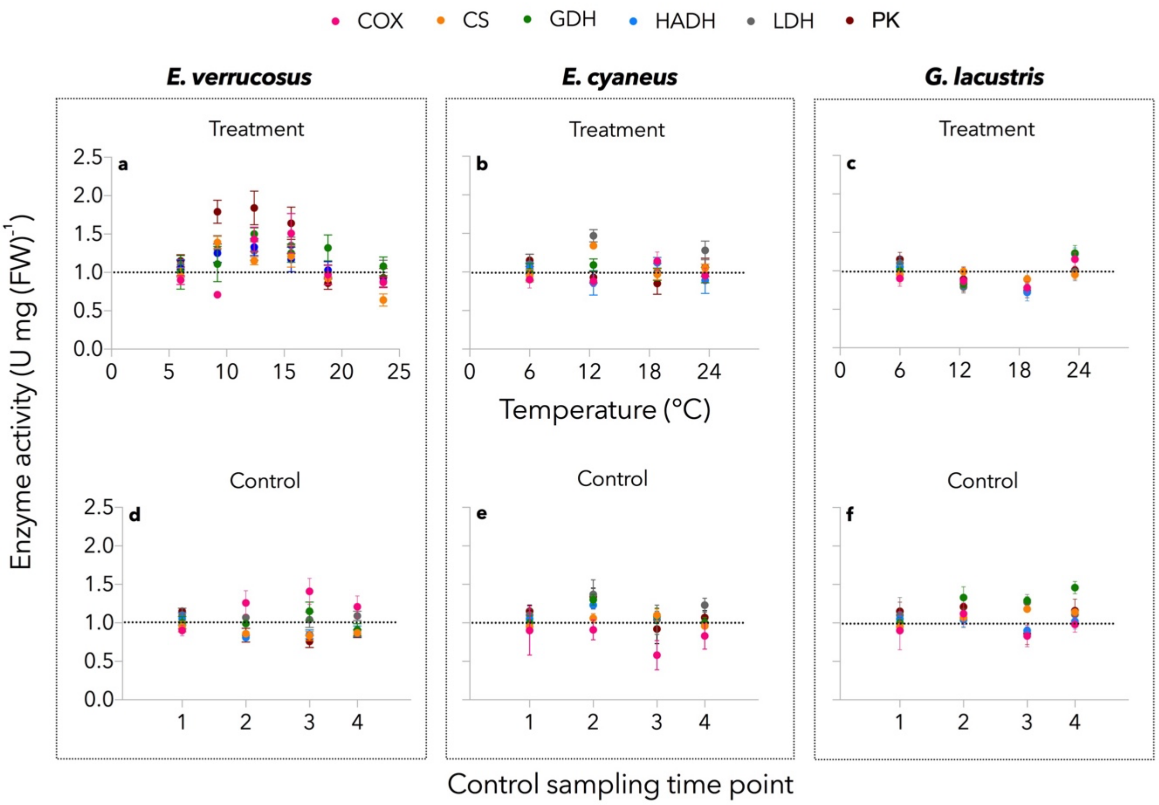

Figure S2: Maximum enzyme activities of cytochrome-c-oxidase (COX), citrate synthase (CS), glutamate dehydrogenase (GDH), 3-hydroxyacyl-CoA dehydrogenase (HADH), lactate dehydrogenase (LDH), and pyruvate kinase (PK) extracted from tissues of *E. verrucosus* (a), *E. cyaneus* (b) and *G. lacustris* (c) exposed to gradual temperature increase (0.8 °C d^{-1}). Samples of all three species were taken at 6 °C, 12.4°C, 18.8 °C, and 23.6 °C; in addition, samples of *E. verrucosus* were taken at 9.2 °C and 15.6 °C. Controls (d-f) were constantly kept at 6 °C and sampled in parallel with the thermal increase experiment. All measurements were performed at 18.8 °C. Data are presented as means ± s.e.m. (n = 4-7). All enzyme activities are scaled to the respective start value. Dotted horizontal lines indicate start control levels.

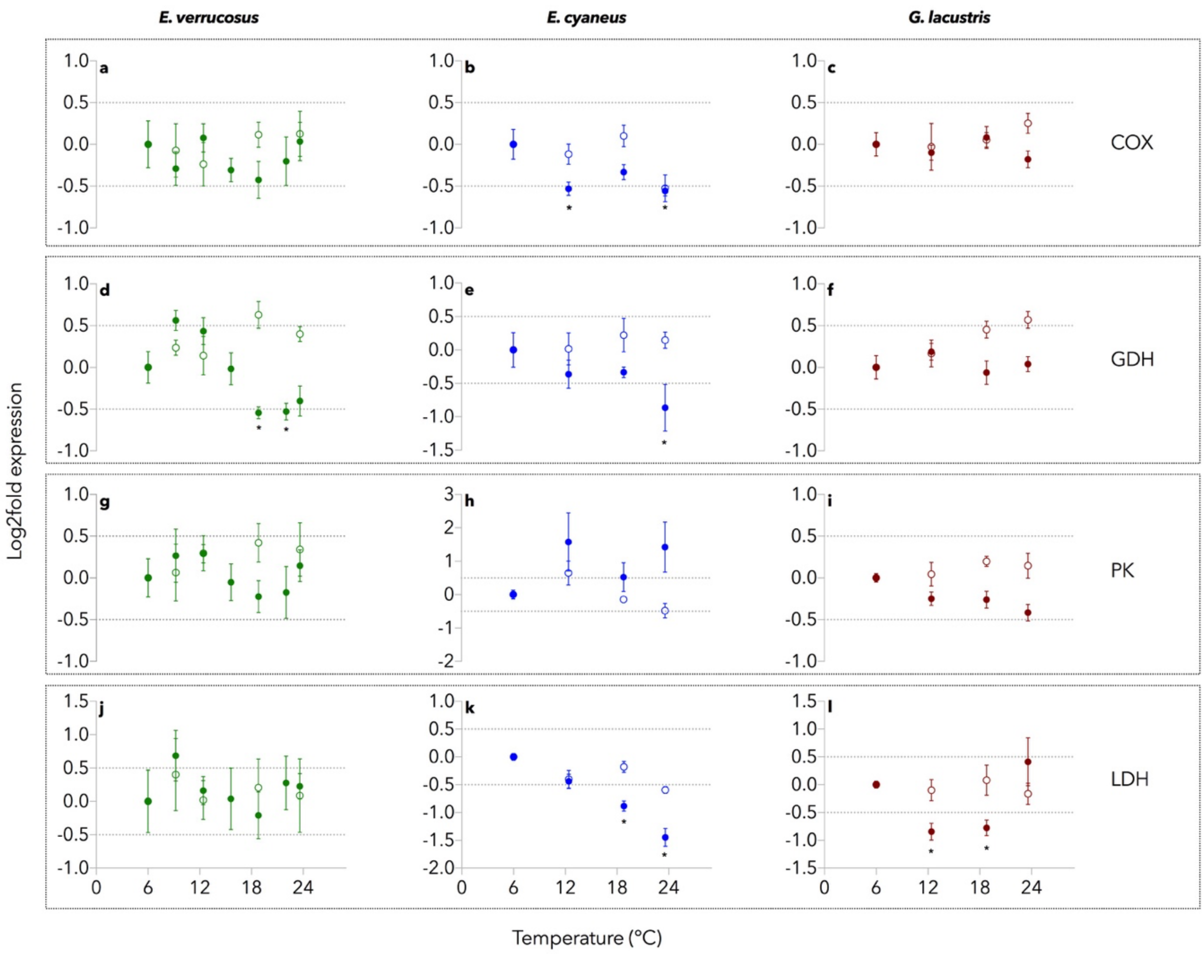

Figure S3: Expression of RNA transcripts of cytochrome-c-oxidase (COX), glutamate dehydrogenase (GDH), pyruvate kinase (PK), and lactate dehydrogenase (LDH) in *E. verrucosus*, *E. cyaneus* and *G. lacustris* exposed to gradual temperature increase (0.8 °C d^{-1}). Samples of all three species were taken at 6 °C, 12.4 °C, 18.8 °C, and 23.6 °C; besides, samples of *E. verrucosus* were taken at 9.2 °C, 15.6 °C, and 22 °C. Expression data are expressed as log2fold change calculated against the same endogenous controls (*actin* and *gapdh*) for all three species. Temperature treated groups are shown with filled circles, whereas the time control groups (kept at 6 °C) are represented by open circles. Asterisks indicate data points outside the range of biological insignificance (± 0.5 log2fold change; dotted horizontal lines), which are significantly different from the 6 °C treatment group (ANOVA, $p < 0.05$). Data are presented as means ± s.e.m. (n = 5-7).

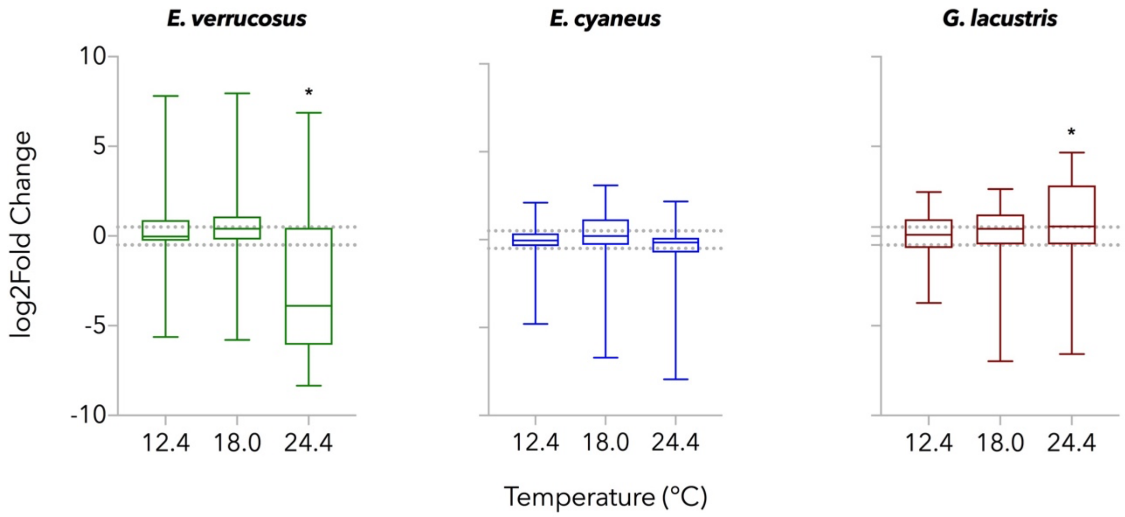

Figure S4: Box-Whisker-Plot showing the abundance of transcripts annotated as ribosomal protein genes. Log2fold changes of ribosomal protein gene expression in *E. verrucosus*, *E. cyaneus* and *G. lacustris* exposed to gradual temperature increase (0.8 °C d^{-1}) relative to the initial control (at 6 °C). Samples were taken at 12.4 °C, 18.0 °C, and 24.4 °C. Asterisks indicate data points outside the range of biological insignificance (± 0.5 log2fold change; dotted horizontal lines) which are significantly different from zero (ANOVA, $p < 0.05$).

Table S1: Summary of enzyme assays.

The given amounts relate to the final volume of 200 µL. Assays were conducted at the respective sampling temperature and at temperatures of 6 °C, 18.8 °C and 23.6 °C. Buffer pH values were adjusted to the respective measuring temperatures.

**Enzyme**	**Physiological function (localization)**	**Components of enzyme assays**	**Start reagent**	**Wave length (nm)**
Cytochrome-c-oxidase (COX)	Final enzyme in the respiratory electron transport chain (inner mitochondrial membrane)	- Tris HCl (20 mM, pH 8.0)<br>- Tween (0.5%)	Cytochrome c (reduced) (50 µM)	550
Citrate synthase (CS)	Pace-making enzyme in the first step of the citric acid cycle, often used as a marker for intact mitochondria and aerobic capacity (mitochondrial matrix)	- Tris HCl (75 mM pH7.6)<br>- Acetyl-CoA (0.4 mM)<br>- 5,5'-Dithiobis-(2-nitrobenzoicacid) (0.25 mM)	Oxaloacetate (0.5 mM)	412
Lactate dehydrogenase (LDH)	Catalyzes the final step in anaerobic glycolysis (cytosol)	- Phosphate buffer (80mM, pH 7.0)<br>- NADH (0.2 mM)	Sodium pyruvate (0.5 mM)	340
Pyruvate kinase (PK)	Catalyzes the final step in the glycolytic pathway (cytosol)	- Tris HCl (128 mM, pH 7.5)<br>- $MgSO_4$ (4.5 mM)<br>- KCl (72 mM)<br>- ADP (5mM)<br>- LDH (1 µg)<br>- NADH (0.2 mM)	Phosphoenol pyruvate (1mM)	340
3-Hydroxyacyl-CoA dehydrogenase (HADH)	Catalyzes the second oxidation step of fatty acid catabolism (mitochondrial matrix)	- Imidazole HCl (50mM, pH 6.6)<br>- Antimycin (0.5 µg)<br>- NADH (0.15 mM)	Aceto acetyl-CoA (0.1 mM)	340
Glutamate dehydrogenase (GDH)	Represents a key link between catabolic and metabolic pathways (mitochondrial matrix)	- Phosphate buffer (80mM, pH 8.0)<br>- NH4 acetate (100 mM)<br>- NADH (0.2 mM)<br>- ADP (1mM)	α-Ketoglutarate (5mM)	340

Table S2: Equations of models fitted to data presented in Figure 2 (Dynamic Fitting; Sigma Plot 13).

Data location	Equation category	Equation
Fig. 2a (*E. verrucosus*, green)	Lognormal; peak; 4 parameter	$y = y0 + \frac{a}{x}\exp\left[-0.5\left(\frac{\ln\left(\frac{x}{x0}\right)}{b}\right)^2\right]$
Fig. 2b (*E. verrucosus*, green)	Lorentzian; peak; 3 parameter	$y = \frac{a}{1+\left(\frac{x-x_0}{b}\right)^2}$
Fig. 2c-e (*E. verrucosus*, green)	Lognormal; peak; 3 parameter	$y = \frac{a}{x}\exp\left[-0.5\left(\frac{\ln\left(\frac{x}{x0}\right)}{b}\right)^2\right]$
Fig. 2a-f (*E. cyaneus*, blue and *G. lacustris*, red)<br>Fig. 2f (*E. verrucosus*, green)	Exponential growth; 2 parameter	$y = a\exp^{bx}$

Table S3: Thermal sensitivity of key metabolic enzymes.

Q_{10}-values (6-18.8 °C) of enzymatic activities of cytochrome-c-oxidase (COX), citrate synthase (CS), glutamate dehydrogenase (GDH), lactate dehydrogenase (LDH), 3-hydroxyacyl-CoA dehydrogenase (HADH) and pyruvate kinase (PK) in dependence of sampling temperature (T (°C)). Amphipods were sampled along an experimental thermal gradient and temperature was increased by 0.8 °C per day starting at 6 °C. Different letter indicate statistical differences between the Q_{10}-values within a species and for a certain enzyme; ANOVA < 0.05.

**Species**	**Enzyme**	**T (°C)**	**Mean Q_{10}**	**s.e.m.**
E. verrucosus	COX	6.00	1.34 ^{A}	0.14
E. verrucosus	COX	9.20	1.76 ^{A}	0.03
E. verrucosus	COX	12.40	2.62 ^{B}	0.22
E. verrucosus	COX	15.60	2.01 ^{AB}	0.16
E. verrucosus	COX	18.80	1.58 ^{A}	0.17
E. verrucosus	COX	23.60	1.70 ^{A}	0.21
E. verrucosus	CS	6.00	1.81 ^{A}	0.06
E. verrucosus	CS	9.20	1.92 ^{A}	0.04
E. verrucosus	CS	12.40	1.82 ^{A}	0.04
E. verrucosus	CS	15.60	1.88 ^{A}	0.04
E. verrucosus	CS	18.80	1.72 ^{A}	0.22
E. verrucosus	CS	23.60	2.08 ^{A}	0.05
E. verrucosus	GDH	6.00	2.31 ^{A}	0.11
E. verrucosus	GDH	9.20	2.00 ^{A}	0.06
E. verrucosus	GDH	12.40	2.33 ^{A}	0.12
E. verrucosus	GDH	15.60	1.85 ^{A}	0.08
E. verrucosus	GDH	18.80	2.12 ^{A}	0.10
E. verrucosus	GDH	23.60	2.20 ^{A}	0.09
E. verrucosus	HADH	6.00	2.00 ^{A}	0.07
E. verrucosus	HADH	9.20	1.79 ^{A}	0.06
E. verrucosus	HADH	12.40	2.31 ^{A}	0.22
E. verrucosus	HADH	15.60	1.78 ^{A}	0.21
E. verrucosus	HADH	18.80	1.77 ^{A}	0.14
E. verrucosus	HADH	23.60	2.15 ^{A}	0.13
E. verrucosus	LDH	6.00	2.41 ^{A}	0.13
E. verrucosus	LDH	9.20	2.29 ^{A}	0.19
E. verrucosus	LDH	12.40	2.54 ^{A}	0.03
E. verrucosus	LDH	15.60	2.00 ^{A}	0.12

E. verrucosus	LDH	18.80	2.26 [A]	0.07
E. verrucosus	LDH	23.60	2.45 [A]	0.12
E. verrucosus	PK	6.00	2.17 [A]	0.64
E. verrucosus	PK	9.20	4.04 [ABC]	0.10
E. verrucosus	PK	12.40	4.93 [B]	0.44
E. verrucosus	PK	15.60	4.42 [BC]	0.15
E. verrucosus	PK	18.80	2.75 [AC]	0.48
E. verrucosus	PK	23.60	3.40 [ABC]	0.39
E. cyaneus	COX	6.00	1.65 [A]	0.11
E. cyaneus	COX	12.40	2.03 [A]	0.04
E. cyaneus	COX	18.80	1.80 [A]	0.12
E. cyaneus	COX	23.60	1.80 [A]	0.04
E. cyaneus	CS	6.00	1.88 [A]	0.09
E. cyaneus	CS	12.40	1.86 [A]	0.07
E. cyaneus	CS	18.80	1.75 [A]	0.04
E. cyaneus	CS	23.60	1.99 [A]	0.09
E. cyaneus	GDH	6.00	2.07 [A]	0.05
E. cyaneus	GDH	12.40	2.02 [A]	0.12
E. cyaneus	GDH	18.80	2.08 [A]	0.08
E. cyaneus	GDH	23.60	2.31 [A]	0.13
E. cyaneus	HADH	6.00	1.82 [A]	0.08
E. cyaneus	HADH	12.40	1.81 [A]	0.13
E. cyaneus	HADH	18.80	2.09 [A]	0.09
E. cyaneus	HADH	23.60	2.27 [A]	0.06
E. cyaneus	LDH	6.00	2.39 [A]	0.19
E. cyaneus	LDH	12.40	2.76 [A]	0.15
E. cyaneus	LDH	18.80	2.10 [A]	0.12
E. cyaneus	LDH	23.60	2.22 [A]	0.13
E. cyaneus	PK	6.00	2.91 [A]	0.42
E. cyaneus	PK	12.40	4.41 [A]	0.33
E. cyaneus	PK	18.80	1.70 [A]	0.32
E. cyaneus	PK	23.60	2.62 [A]	0.53
G. lacustris	COX	6.00	1.78 [A]	0.31
G. lacustris	COX	12.40	1.90 [A]	0.06
G. lacustris	COX	18.80	1.62 [A]	0.15

G. lacustris	COX	23.60	2.02 [A]	0.15
G. lacustris	CS	6.00	1.75 [A]	0.12
G. lacustris	CS	12.40	1.81 [A]	0.06
G. lacustris	CS	18.80	1.93 [A]	0.07
G. lacustris	CS	23.60	1.76 [A]	0.05
G. lacustris	GDH	6.00	1.95 [A]	0.09
G. lacustris	GDH	12.40	2.10 [A]	0.08
G. lacustris	GDH	18.80	2.00 [A]	0.08
G. lacustris	GDH	23.60	2.31 [A]	0.12
G. lacustris	HADH	6.00	2.01 [A]	0.17
G. lacustris	HADH	12.40	2.14 [A]	0.09
G. lacustris	HADH	18.80	1.51 [A]	0.19
G. lacustris	HADH	23.60	2.00 [A]	0.08
G. lacustris	LDH	6.00	1.72 [A]	0.11
G. lacustris	LDH	12.40	2.02 [A]	0.14
G. lacustris	LDH	18.80	1.85 [A]	0.14
G. lacustris	LDH	23.60	1.92 [A]	0.1
G. lacustris	PK	6.00	6.66 [A]	1.63
G. lacustris	PK	12.40	6.28 [A]	1.2
G. lacustris	PK	18.80	4.88 [A]	0.48
G. lacustris	PK	23.60	7.25 [A]	2.02
